# Genome-wide in silico identification of glutathione S-transferase (GST) gene family members in fig (*Ficus carica* L.) and expression characteristics during fruit color development

**DOI:** 10.7717/peerj.14406

**Published:** 2023-01-25

**Authors:** Longbo Liu, Shuxuan Zheng, Dekun Yang, Jie Zheng

**Affiliations:** 1 School of Life Science, Huaibei Normal University, Huaibei, Anhui, China; 2 Xiayi Branch of Henan Agricultural Radio and Television School, Shangqiu, Henan, China

**Keywords:** Fig, GST gene family identification, Expression characteristics, Fruit colordevelopment, Anthocyanin content

## Abstract

Glutathione *S*-transferase (GSTs), a large and diverse group of multi-functional enzymes (EC 2.5.1.18), are associated with cellular detoxification, various biotic and abiotic stress responses, as well as secondary metabolites transportation. Here, 53 members of the *FcGST* gene family were screened from the genome database of fig (*Ficus carica*), which were further classified into five subfamilies, and the tau and phi were the major subfamilies. These genes were unevenly distributed over all the 13 chromosomes, and 12 tandem and one segmental duplication may contribute to this family expansion. Syntenic analysis revealed that FcGST shared closer genetic evolutionary origin relationship with species from the *Ficus* genus of the *Moraceae* family, such as *F. microcarpa* and *F. hispida*. The FcGST members of the same subfamily shared similar gene structure and motif distribution. The *α* helices were the chief structure element in predicted secondary and tertiary structure of FcGSTs proteins. GO and KEGG indicated that FcGSTs play multiple roles in glutathione metabolism and stress reactions as well as flavonoid metabolism. Predictive promoter analysis indicated that *FcGSTs* gene may be responsive to light, hormone, stress stimulation, development signaling, and regulated by MYB or WRKY. RNA-seq analysis showed that several *FcGSTs* that mainly expressed in the female flower tissue and peel during ‘Purple-Peel’ fig fruit development. Compared with ‘Green Peel’, *FcGSTF1*, and *FcGSTU5*/*6*/*7* exhibited high expression abundance in the mature fruit purple peel. Additionally, results of phylogenetic sequences analysis, multiple sequences alignment, and anthocyanin content together showed that the expression changes of *FcGSTF1*, and *FcGSTU5*/*6*/*7* may play crucial roles in fruit peel color alteration during fruit ripening. Our study provides a comprehensive overview of the *GST* gene family in fig, thus facilitating the further clarification of the molecular function and breeding utilization.

## Introduction

The glutathione *S*-transferase (*GST*) gene family is an ancient and multigene group that is found in almost all living organisms. Two binding domains, the glutathione binding site (G-site) in the N-terminal (GST-N) and the substrate binding site (H-site) in the C-terminal, are well conserved in typical GST enzymes (Edwards & Dixon, 2005). The main function of GSTs is to catalyze the conjugation of an array of electrophilic compounds of exogenous and endogenous origins to reduced glutathione (GSH) (Cummins et al., 2011).

Recent advances in whole-genome sequencing and genome-wide analysis have led to the identification and characterization of *GST* gene families in diverse plant species. Research results show that because it is a multigene family, the *GST* family members have different numbers of genes. In *Arabidopsis*, a total of 53 *GST* family members have been identified (Sappl et al., 2004). Seventy-nine members were found in rice (Jain, Ghanashyam & Bhattacharjee, 2010), 59 in *Gossypium raimondii*, 49 in *G. arboreum* (Dong et al., 2016), 90 in tomato (Islam et al., 2017), 54 in peach (Zhao et al., 2017), 69 in apple (Jiang et al., 2019), 97 in kiwifruit (Liu et al., 2019b), 74 in tea (Liu et al., 2019a), 85 in pepper (Islam et al., 2019), 179 in *Brassica napus* (Wei et al., 2019b), 130 in cultivated strawberry (*Fragaria* × *ananassa*) (Lin et al., 2020), 49 in melon (Wang et al., 2020), 92 in *Medicago truncatula* (Hasan et al., 2021), 39 in Hami melon (Song et al., 2021a), and 57 in pear (Li et al., 2022). Although the *GST* gene family has been extensively characterized in various plant species, its exploration has been limited in fig.

According to the gene structure, protein sequence similarity, gene function, and immunological characteristics within the family, the *GST* gene family can be further categorized into 14 subfamilies. Of these, four subfamilies (tau (U), phi (F), lambda (L), and TCHQD) are unique to the plant (Lallement et al., 2014). As an ancient protein superfamily with multipurpose roles, *GST* genes have diverse functions in plants (Vaish et al., 2020). Previous studies have emphasized the detoxification roles of GSTs *via* transformation, conjugation, and compartmentation phases, which thereby decrease toxic damage (Edwards & Dixon, 2005). They also showed the widely indispensable regulatory functions when plants encounter various stressors, such as cold (Kayum et al., 2018; Song et al., 2021a), salinity (Xu et al., 2016), heavy metals (Gao et al., 2020; Jing et al., 2021), drought (Chen et al., 2012; Xu et al., 2016), and chemical toxicity (Xu et al., 2016). Another outstanding role of several *GST* genes is governing the sequestration of secondary metabolic compounds, such as flavonoids, anthocyanin, proanthocyanidins (PAs), and porphyrins (Dixon, Skipsey & Edwards, 2010; Liu et al., 2019a). Other studies have indicated that GST genes play important roles in coloration. Several GSTF subfamily genes are required for anthocyanin transport and accumulation, such as *TT19* (GSTF12) in *Arabidopsis* (Kitamura, Shikazono & Tanaka, 2003; Sun, Li & Huang, 2012), *An9* in petunia (Mueller et al., 2000), *CkmGST3* in cyclamen (Kitamura et al., 2012), *DcGSTF2* in carnations (Sasaki et al., 2012), *LcGST4* in litchi (Hu et al., 2016), *FvRAP* in strawberry (Luo et al., 2018), *CsGSTa* in tea (Liu et al., 2019a), *CsGSTF1* in purple tea (Wei et al., 2019a), *AcGST1* in kiwifruit (Liu et al., 2019b), *MdGSTF6* in apple (Jiang et al., 2019), *IbGSTF4* in sweet potato (Kou et al., 2019), *PpGST1* in peach (Zhao et al., 2020), *GST1* in Japanese gentian (Tasaki et al., 2020), *GhGSTF12* in cotton (Shao et al., 2021), *LhGST* in lilies (Cao et al., 2021), and *PcGST57* in pear (Li et al., 2022). Several members of the GSTU subfamily are also involved in the regulation of anthocyanin accumulation, including *Bz2* in maize (Marrs et al., 1995), *CsGSTb* and *CsGSTc* in tea (Liu et al., 2019a), and *MdGSTU12* in apple (Zhao et al., 2021). Although it is known that GST is involved in anthocyanin accumulation in many species, there is a lack of information about GST genes’ role in fig fruit color formation.

Fig (*Ficus carica* L.), belonging to the *Moraceae* family, originated in western Asia, and is one of the earliest domesticated fruit crops. Ripe fruits contain a variety of beneficial bioactive ingredients, such as dietary fiber, sugars, minerals, carotenoids, and anthocyanins (Wang et al., 2021). The common fig can be classified into green, yellow, and red cultivar categories according to the different coloration levels caused by anthocyanin content in the fruit peel. Anthocyanin content in fig contributes to its nutritional quality and attractiveness, with red fruit having a high market value. To evaluate the potential roles of *GST* in anthocyanin accumulation, we carried out comprehensive identification and expression characteristic analysis of the *GST* gene family in fig. In this study, we used the public high-quality reference genome sequence of fig (Usai et al., 2020) to identify a total of 53 *GST* gene members. Gene features of *FcGSTs* were determined based on bioinformatics analysis, and we found that *FcGSTF1* and *FcGSTU5/6/7* may contribute to fruit peel color. Our results may facilitate the further functional investigation of *FcGSTs*’ role in anthocyanin accumulation in fig.

## Material & Methods

### *FcGST* candidate gene identification

Genome sequences and annotation information of fig (*Ficus carica* ‘Dottato’) were collected from the National Center for Biotechnology Information (NCBI) (https://www.ncbi.nlm.nih.gov/genome/?term=Ficus+carica) (Usai et al., 2020). The protein sequences of the fig genome were predicted using the Batch translator CDS to protein function of TBtools (Chen et al., 2020) and by protein sequences of fig obtained from EnsemblPlants (http://ftp.ebi.ac.uk/ensemblgenomes/pub/release-54/plants/fasta/ficus_carica/pep/). The protein sequences of 53 AtGSTs in *Arabidopsis* were downloaded from TAIR (https://www.arabidopsis.org/browse/genefamily/gst.jsp) (Sappl et al., 2004). The protein sequences of 79 OsGSTs in rice were obtained from the Rice Genome Annotation Project Database (RGAP) (http://rice.uga.edu/downloads_gad.shtml) according gene id (Jain, Ghanashyam & Bhattacharjee, 2010), but the protein sequences of OsGSTU3 (LOC_Os10g38501) and OsGSTU4 (LOC_Os10g38495) were not found in RGAP. Therefore, a total of 130 sequences were taken as queries in the local BLASTP search against the protein sequences of fig to retrieve all *GST* family candidates with *E*-value (≤ e ^−10^) and identify ≥ 50% as thresholds. Moreover, the hidden Markov model (HMM) profiles of the GST-N (PF02798) and GST-C (PF00043) domain, obtained from the Pfam database (http://pfam.xfam.org/), were also employed as queries to search the FcGST candidates using HMMER 3.1 with a cut off *E*-value (≤ e ^−5^). After redundant sequences were removed, the rest FcGST candidates were validated using SMART (http://smart.embl-heidelberg.de/) and NCBI conserved domain database (CDD) (https://www.ncbi.nlm.nih.gov/Structure/cdd/wrpsb.cgi) to examine the presence of the conserved domains with the default parameters. The protein sequences and gene id of 53 AcGSTs, 77 OsGSTs, and 53 FcGSTs are slisted in Data S1.

### Phylogenetic analysis and subfamily categorization

Multiple-sequence alignments of GST protein sequences from *Arabidopsis*, rice, and fig were performed using the default settings of Clustal Omega (https://www.ebi.ac.uk/Tools/msa/clustalo/). A phylogenetic tree was generated using the default parameters of neighbor-joining method in MEGA X software with 1,000 bootstrap replicates (http://www.megasoftware.net/). According to the classification records of subfamily members in *Arabidopsis* and rice, these FcGST genes were further categorized into five different subfamilies. To precisely predict their molecular function, GST genes related to anthocyanin accumulation were used to construct a phylogenetic tree with 53 FcGST genes via MEGA X software according to the procedures mentioned above.

The length of cDNA and CDS were retrieved from fig genome annotation data. Physicochemical parameters (such as polypeptide length, molecular weight, and isoelectric point) of FcGST proteins were evaluated using the ExPasy website (http://web.expasy.org/protparam/). The online soft tools of PSORT (http://psort.hgc.jp/form.html) and BUSCA (http://busca.biocomp.unibo.it/) were used to predict these FcGSTs subcellular localization.

### Chromosomal locations and collinearity analysis

A genome annotation file was used to collect 53 *FcGST* genes’ chromosomal location information. The positions of the *FcGSTs* were plotted to chromosomes using TBtools, and only gene matched chromosomes were shown (Chen et al., 2020).

The genome data of *Malus domestica* and *Prunus persica* were downloaded from Genome Database for Rosaceae (GDR) (https://www.rosaceae.org). The genome data of *Vitis vinifera* were obtained from EnsemblPlants (http://ftp.ebi.ac.uk/ensemblgenomes/pub/release-52/plants/fasta/vitis_vinifera/). The genome data of *Ficus hispida* and *F. microcarpa* were collected from the database of the National Genomics Data Center (NGDC) with BioProject number PRJCA002187. The collinear relationships among the *FcGST* genes in fig and *A. thaliana*, rice, apple, peach, and grape were performed using the Multiple Collinearity Scan toolkit (MCScanX) with default parameters (Wang et al., 2012). The collinear relationships among *F. hispida*, *F. microcarpa*, and fig were also analyzed using the default parameters of MCScanX. Two or more genes located within the present 100 kb region on the same chromosome were defined as a tandem repeat event, while those located beyond the 100 kb region were considered a segmental duplication event. The syntenic relationship was presented as circle plot using TBtools (Chen et al., 2020). Sequence similarity analysis of the duplicated gene pairs used the default settings of Clustal Omega (https://www.ebi.ac.uk/Tools/msa/clustalo/).

### Gene structure, protein motif analysis, and protein structure analysis

Exon-intron structure information of *FcGSTs*, *AtGSTs*, *OsGSTs*, *MdGSTs*, *MtGSTs*, *SlGSTs*, and *CmGSTs* were obtained from the genome annotation files of fig, *Arabidopsis* (Sappl et al., 2004), rice (Jain, Ghanashyam & Bhattacharjee, 2010), apple (Jiang et al., 2019), *Medicago truncatula*, (Hasan et al., 2021), tomato (Islam et al., 2017), and melon (Wang et al., 2020), respectively. The conserved motifs sequence and type were analyzed using MEME online server Version 5.4.1 (http://meme-suite.org/tools/meme), and the parameters were set as follows: motif site distribution = any number of repetitions (anr); maximum motif number = 10; and motif length = 6-200. The results of gene structure and motif organization were grouped by phylogenetic tree and displayed using TBtools (Chen et al., 2020). The putative secondary structure of FcGSTs were predicted by NPS@: SOPMA (https://npsa-prabi.ibcp.fr/cgi-bin/npsa_automat.pl?page=npsa%20_sopma.html). The tertiary models of selected FcGST proteins were predicted using ExPaSy Swiss-Model online software (http://swissmodel.expasy.org) and the results were validated by SAVES v6.0 online server (http://services.mbi.ucla.edu/SAVES/). The 3-D structures were visualized by VMD software V1.9.3 (https://www.ks.uiuc.edu/Research/vmd/vmd-1.9.3/).

### Functional analysis

GO annotation analysis of all fig protein sequences was performed using BLASTP with Swiss-UniProt database with *E*-value (≤ e ^−5^). KEGG annotation analysis was performed using the KofamKOALA online program with default parameters (https://www.genome.jp/tools/kofamkoala/). TBtools was used to obtain the GO and KEGG enrichment terms with corrected *p*-value (≤ 0.05) and to plot the enrichment results (Chen et al., 2020). A protein-protein interaction (PPI) network of 53 FcGST proteins was generated using STRING database V11.5 (https://cn.string-db.org/) (Szklarczyk et al., 2021) and visualized in Cytoscape 3.6.1 software program (Shannon et al., 2003).

### *Cis*-acting element analysis of *FcGST* genes

For *cis*-element analysis, the putative promoter sequences (2,000 bp sequence upstream of the translation site) were acquired from all *FcGST* genomic DNA sequences using TBtools (Chen et al., 2020). The putative *cis*-acting elements were predicted using PlantCARE program with default parameters (http://bioinformatics.psb.ugent.be/webtools/plantcare/html/).

### RNA-seq data analysis

Three published raw RNA-seq data downloaded from the NCBI Sequence Read Archive (SRA) database (https://www.ncbi.nlm.nih.gov/sra/) were mined to investigate the expression characteristics of *FcGST* genes during fig fruit development. In common purple-peel fig cultivar ‘Zibao’, the development process is divided into six stages. Stages 1 and 2 belong to a rapid growth stage. In stages 3 and 4, fruit size and hardness remain almost unchanged. Stages 5 and 6 belong to the fruit mature stage, where stage 5 corresponds to commercial ripeness (Zhai et al., 2021). The expression patterns of *FcGSTs* were analyzed at six fruit development stages of the internal female flower (F1-F6) and syconia peel (P1-P6) (accession number SRP315833) (Zhai et al., 2021). RNA-seq data of ‘Green Peel’ and its bud mutation cv. ‘Purple Peel’ at young and mature fruit development stages were used to estimate the expression level of *FcGSTs* in peel (accession number SRP114533) (Wang et al., 2017). Unlike typical climacteric fruit, such as banana, figs cannot ripen by themselves or with exogenous treatment once harvested before commercial ripeness stage. Ethephon treatment has been reported to stimulate fig fruit growth and shorten the ripening period with no negative effect on fruit quality. Ethephon solution was injected through the fruit ostiole in order to determine the role of ethylene in fig-fruit ripening using RNA sequencing (Cui et al., 2020). Transcriptome data of fig fruit after ethephon application at two, four, and six days were used to investigate the *FcGST* gene expression profile in ethylene-regulated pigmentation changes of fig female flowers (accession number SRP249501) (Cui et al., 2020).

The quality of raw reads were checked using FastQC (version 0.11.8, https://www.bioinformatics.babraham.ac.uk/projects/fastqc/). After adapter removal by Trimmomatic (Bolger, Lohse & Usadel, 2014), the clean reads were mapped to fig genome data by HISAT2 with default parameters (Kim et al., 2019). The transcript abundance of each sample was calculated by StringTie Quantify (Pertea et al., 2015). The transcripts per kilobase of exon model per million mapped reads (TPM) was applied to describe the gene transcript level. The TPM value was transformed into log_2_ (TPM + 1) in the presented heatmaps. DESeq2 R package was used to identify the differential expression genes (DEGs, —log_2_(fold change)— ≥ 1 and *p*-adjust <0.05) (Love, Huber & Anders, 2014). During ‘Zibao’ fig fruit ripening, DEGs were identified across different flower and peel development stages and compared with stage 1 (F1 or P1). Gene expression heatmaps were plotted using TBtools (Chen et al., 2020).

### Plant material

We used two common fig cultivars ‘Bojihong’ and ‘Orphan’, planted at the horticultural experimental station of Huaibei Normal University, Huaibei, China (33°59′N, 116°48′E). These fig trees were 4 years old with 3 m × 3 m spacing and standard cultivation. Fig fruits were harvested at 30, 60, and 70 days after fruit setting. Each stage contained three replicates and each replicate randomly sampled at least 20 healthy and uniform fruits. After cleaning, fig fruit peels on the middle site were isolated with receptacles using a scalpel. All samples were immediately frozen in liquid nitrogen and stored at −80 °C.

### Measurement of anthocyanin content

The total anthocyanin content of fig fruit peel was measured according to Li, An & Wang (2020). After approximately 0.5 g of fig peel was incubated in 5 ml extraction solution (1% (v/v) HCl-methanol) for 24 h at room temperature under shading, the supernatant was centrifuged at 8,000 *g* for 15 min, and the absorbance at 530, 620, and 650 nm was measured. The anthocyanin content was calculated according to the following formula: content (nmol g^−1^ FW) = [(OD530 − OD620) − 0.1 × (OD650 − OD620)]/ *ɛ* × V/M × 10^6^, V represents the volume of extraction solution; M represents the weight of each sample, and the absorbency index of total anthocyanin is 4.62 × 10^4^. Three independent replicates were used for mean value calculation.

### Quantitative real-time PCR (qRT-PCR) analysis

As for qRT-PCR assay, total RNA extraction from the fig fruit peel of three biological replicates per sample was carried out using the CTAB method. We used the One-Step gDNA Removal and cDNA Synthesis Supermix kit (Transgen Biotech, Peking, China) to reverse transcribe the total RNA. We added 20-µL of reaction mixture to each well, including 1 µL cDNA, 0.5 µL each of 5′ and 3′ specific primer, 10 µL chamQ SYBR qPCR Master Mix (Vazyme, Shanghai, China), and 8 µL DEPC water. The ABI 7300 Real-Time PCR system was employed to perform the qRT-PCR amplification program with the following settings: 95 °C for 5 min, 40 cycles of: 95 °C for 5 s, 60 °C for 35 s. The relative expression of target genes was calculated with the 2^−ΔΔCT^ method (Livak & Schmitten, 2001) and normalized by *FcActin* (Vangelisti et al., 2019). Each sample was quantified with three replicates. All qRT-PCR primers are listed in Data S2.

### Data analysis

Statistical analysis was performed with SPSS 26.0 software. Significance (*p* < 0.05) was evaluated by analysis of variance (ANOVA) and Duncan’s multiple range test. Graphs were generated in Excel 2016.

## Results

### Identification and phylogenetic analysis of *FcGST* genes

Based on 130 AtGSTs and OsGST protein sequences, we obtained 69 putative GST members in the fig genome database using BLASTp assay. According to HMM search, 70 members were identified as putative GST candidates in fig. After removing redundant transcripts and examining two conserved domains, namely, the N-terminal and C-terminal, a total of 53 genes were screened as the putative GST gene family members in fig.

**Figure 1:**
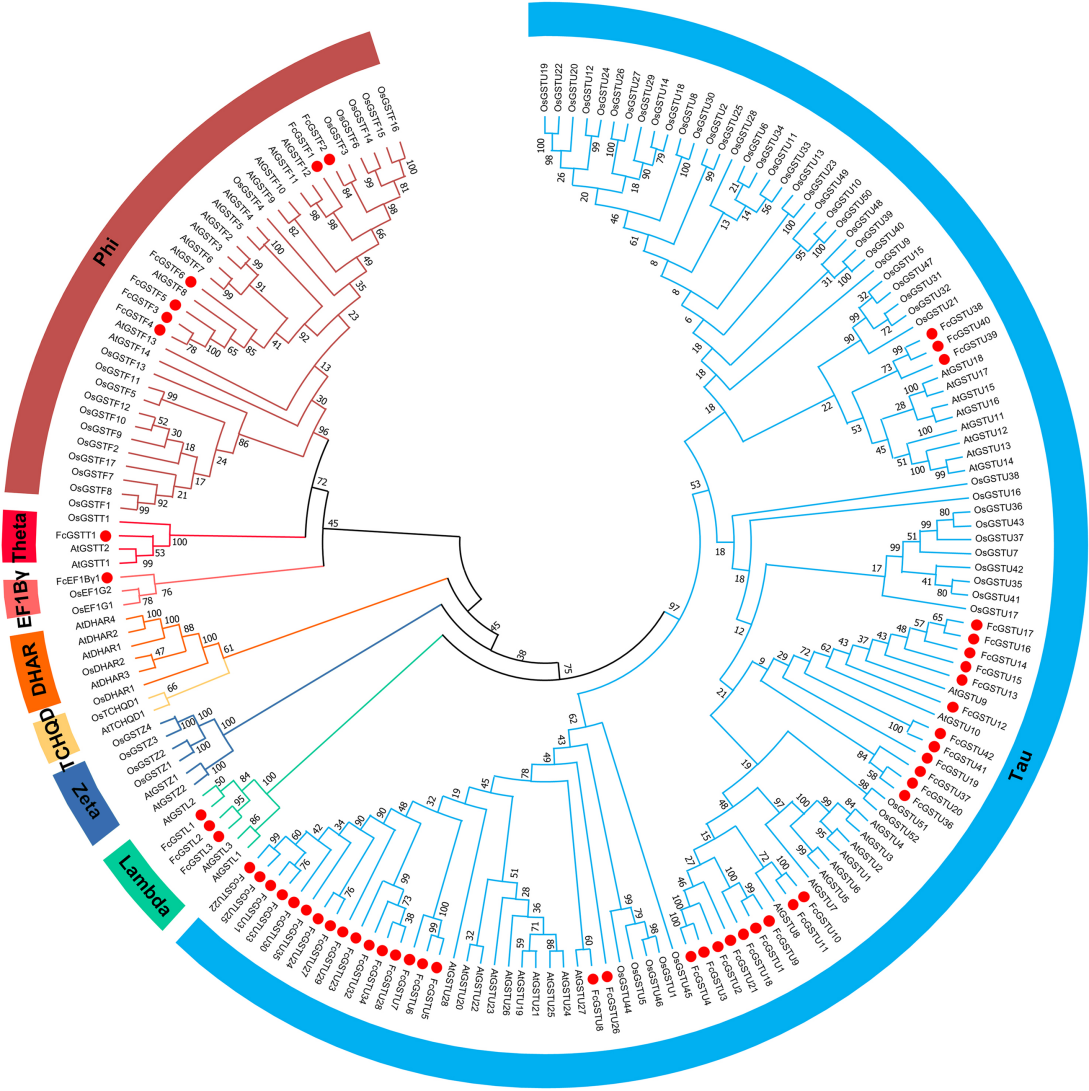
Phylogenetic analysis of GSTs from fig, *Arabidopsis*, and rice. The different color arcs on the periphery of the circle indicate the members of different subfamilies. Red dots highlight the fig *FcGST* genes. The species names are abbreviated as follows: At, *Arabidopsis thaliana*; Os, *Oryza sativa*, and Fc, *Ficus carica*. The phylogenetic tree was constructed using MEGA X program based on the NJ method and 1,000 bootstrap replications.

**Figure 2:**
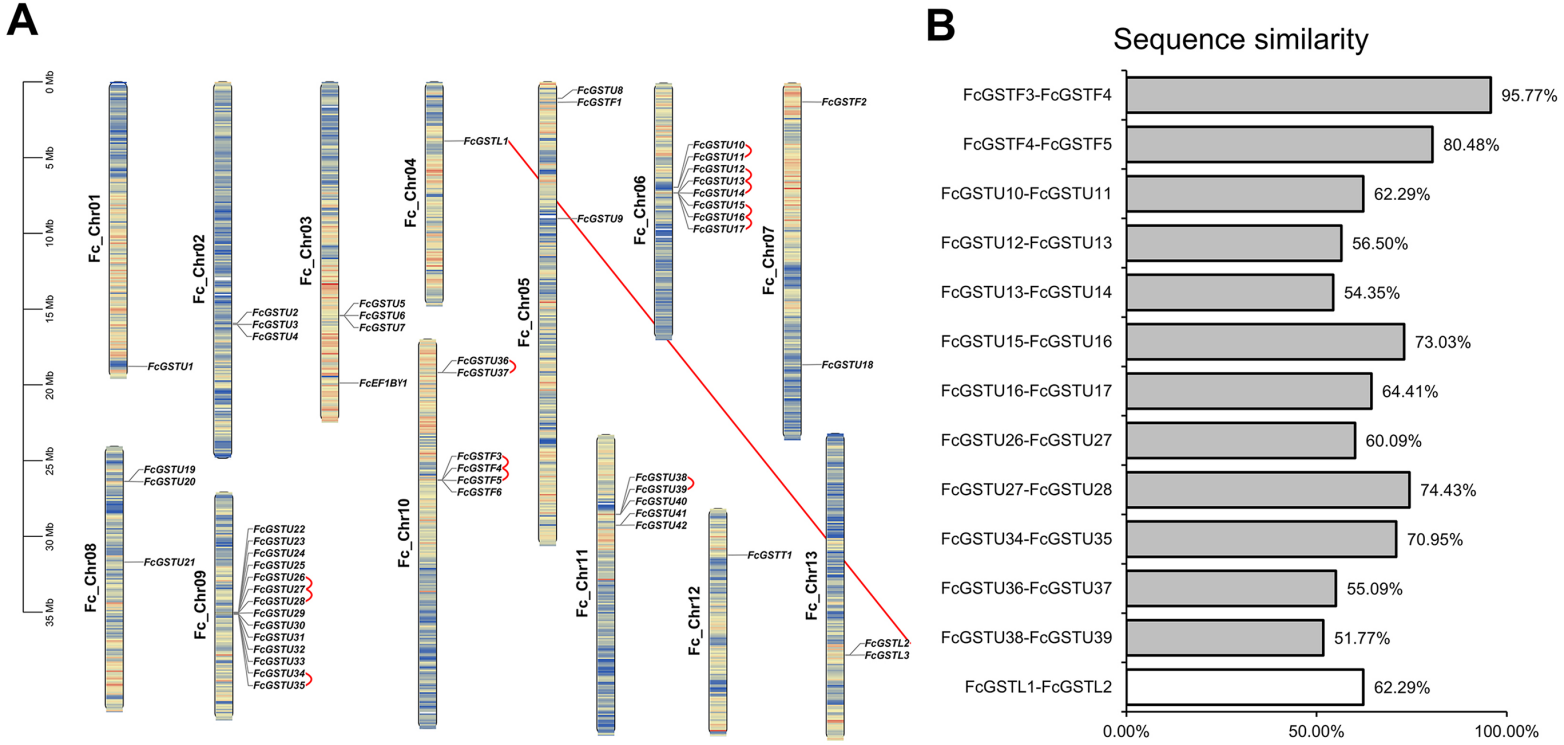
The chromosomal location, synteny and sequence similarity analysis of *FcGST* genes. (A) Chromosome distributions and synteny relationship of 53 *FcGSTs* in the fig genome. Synteny analysis of *FcGST* genes in fig was performed by MCScanX; Red curves indicate tandem duplication gene pairs, and the red solid line indicates the segmental duplication gene pair. (B) Sequence similarity among the synteny gene pairs. The similarity of protein sequence was analyzed by Clustal Omega. Grey rectangles represent tandem duplication, and the white rectangle represent segmental duplication.

To evaluate the evolutionary relationships among the AtGSTs, OsGSTs, and FcGSTs, a phylogenetic tree was constructed using the NJ method of MEGA X. These proteins were classified into eight distinct subfamilies. Tau, lambda, zeta, TCHQD, DHAR, EF1B*γ*, theta, and phi (Fig. 1). Among these subfamilies, the tau and phi groups were found to be the most abundant in *Arabidopsis*, rice, apple, peach, melon, pepper, and fig, accounting for 69.39% (melon)–90.56% (fig) (Data S3). Three FcGST genes were clustered into the lambda subfamily, and only one FcGST gene was categorized as an EF1B*γ* and theta subfamily member. However, no DHAR and TCHQD members were identified in fig. These GST members of fig were named by adding prefix ‘Fc’ (*Ficus carica*) to the subfamily identifiers and their chromosomal order (Figs. 1 and 2).

The gene length of the *FcGST* genes varied from 291 bp (*FcGSTU19*) to 6,368 bp (*FcGSTT1*) (Table 1). The lengths of the coding DNA sequence (CDS) of 34 *FcGSTs* (64.15%) were between 603 (*FcGSTU33*) and 696 bp (*FcGSTU21*). The polypeptide of most FcGSTs were similar, with approximately 43 members (81.12%) ranging from 201 aa to 269 aa. The average molecular weight (MW) of FcGST protein was 25.73 kDa. The change of putative theoretical isoelectric point (pI) varied from 4.71 (FcGSTU34) to 10.38 (FcGSTU19), and 40 members (75.47%) were acidic proteins. Two online soft tools, PSORT and BUSCA, were used to predict FcGST subcellular localization. Most of the FcGST proteins were predicted to be localized in the cytoplasm (43), followed by microbody (26), mitochondria (15), chloroplast (nine), nucleus (seven), plasma membrane (four), and vacuole (one).

**Table 1:** The gene or protein features and subcellular localization prediction of GST members in fig.

Subfamily	Gene name	Gene ID	Gene length (bp)	CDS length (bp)	Protein length (aa)	MW (kDa)	Theoretical pI	Subcellular location
Tau	*FcGSTU1*	FCD_00032091	3194	678	226	26.07	5.67	Cy^a,b^
	*FcGSTU2*	FCD_00019114	2408	660	220	25.29	8.2	Cy^a,b^
	*FcGSTU3*	FCD_00019118	1367	675	225	25.85	5.93	Nu^a^, Cy^a,b^
	*FcGSTU4*	FCD_00019120	1550	675	225	25.83	5.4	Nu^a^, Cy^a,b^
	*FcGSTU5*	FCD_00014951	1315	660	220	25.50	6.25	Cy^a,b^
	*FcGSTU6*	FCD_00014954	1301	654	218	25.36	6.14	Cy^a,b^
	*FcGSTU7*	FCD_00014956	1277	663	221	25.45	6.02	Nu^a^, Cy^a,b^
	*FcGSTU8*	FCD_00001509	1441	666	222	25.11	5.07	Cp^a^, Nu^a^, Cy^a^
	*FcGSTU9*	FCD_00034865	3201	678	226	26.05	5.94	Cy^a,b^
	*FcGSTU10*	FCD_00018160	1035	579	193	22.50	5.46	Cy^a,b^
	*FcGSTU11*	FCD_00018161	1032	666	222	25.73	5.49	Cy^a,b^
	*FcGSTU12*	FCD_00018188	949	675	225	25.75	5.47	Cy^a,b^
	*FcGSTU13*	FCD_00018189	1080	693	231	25.97	7.62	Cy^a,b^
	*FcGSTU14*	FCD_00018190	1187	756	252	28.47	4.97	Cy^a,b^
	*FcGSTU15*	FCD_00018191	1617	537	179	20.52	5.17	Cy^a,b^
	*FcGSTU16*	FCD_00018192	1133	693	231	26.28	6.02	Cy^a,b^
	*FcGSTU17*	FCD_00018193	1272	675	225	25.74	5.49	Cy^a,b^
	*FcGSTU18*	FCD_00025212	1731	681	227	26.31	8.53	Cy^a,b^, Nu^a^
	*FcGSTU19*	FCD_00011212	291	291	97	11.21	10.38	Cp^a^, Nu^a,,b^
	*FcGSTU20*	FCD_00011215	2166	675	225	25.93	6.6	Cy^a,b^
	*FcGSTU21*	FCD_00021672	1405	696	232	27.14	5.57	Cy^a,b^
	*FcGSTU22*	FCD_00016043	919	666	222	25.39	5.78	Cy^a,b^, Cp^a^
	*FcGSTU23*	FCD_00016046	1221	669	223	26.20	8.31	Cy^a^, Cp^b^
	*FcGSTU24*	FCD_00016047	3790	1314	438	48.56	6.31	Cy^a^, Nu^b^
	*FcGSTU25*	FCD_00016048	901	549	183	20.78	6.13	Cy^a,b^
	*FcGSTU26*	FCD_00016050	2051	660	220	25.01	9.78	Nu^a,,b^
	*FcGSTU27*	FCD_00016051	1330	660	220	25.37	5.52	Cp^a^, Cy^a,b^
	*FcGSTU28*	FCD_00016052	1325	690	230	26.93	9.05	Cp^a,,b^
	*FcGSTU29*	FCD_00016055	1803	807	269	30.89	9.65	Cy^a,b^
	*FcGSTU30*	FCD_00016057	1673	660	220	25.39	6.46	Cy^a,b^
	*FcGSTU31*	FCD_00016059	1342	639	213	24.25	6	Nu^a^, Cy^a^
	*FcGSTU32*	FCD_00016062	1182	552	184	21.37	4.92	Cy^a,b^
	*FcGSTU33*	FCD_00016063	1812	603	201	22.58	8.17	Cy^a,b^
	*FcGSTU34*	FCD_00016064	1116	450	150	17.32	4.71	Cy^a,b^
	*FcGSTU35*	FCD_00016065	2148	705	235	27.29	5.93	Cy^a,b^
	*FcGSTU36*	FCD_00000375	1671	510	170	19.41	4.89	Cy^a^, Nu^b^
	*FcGSTU37*	FCD_00000376	1033	669	223	25.60	5.65	Cp^a^, Cy^a,b^
	*FcGSTU38*	FCD_00014609	1988	741	247	27.46	5.83	Cy^a,b^
	*FcGSTU39*	FCD_00014610	429	429	143	15.80	8.79	Nu^a,,b^, Cy^a^,
	*FcGSTU40*	FCD_00014612	1971	666	222	24.48	5.51	Cy^a,,b^
	*FcGSTU41*	FCD_00014715	1101	681	227	25.66	6.4	Cy^a,b^
	*FcGSTU42*	FCD_00014717	1045	735	245	27.67	5.91	Cy^a,b^
Phi	*FcGSTF1*	FCD_00001545	1041	645	215	24.31	5.45	Cy^a,b^, Mt^a^,
	*FcGSTF2*	FCD_00004068	876	666	222	24.86	8.34	Cp^a^, Cy^a^
	*FcGSTF3*	FCD_00006951	2281	642	214	23.99	6.22	Mt^a^, Cp^b^
	*FcGSTF4*	FCD_00006952	2298	642	214	23.81	6.38	Mt^a^, Nu^b^
	*FcGSTF5*	FCD_00006953	2323	666	222	24.90	6.76	Cy^a^, Cp^b^
	*FcGSTF6*	FCD_00006955	1301	672	224	25.17	5.99	Mt^a^, Cp^a,,b^
Lambda	*FcGSTL1*	FCD_00011611	3048	795	265	30.07	5.76	Cp^b^
	*FcGSTL2*	FCD_00005153	3501	711	237	26.91	5.12	Cy^a^, Cp^b^
	*FcGSTL3*	FCD_00005155	3174	717	239	27.52	5.84	Cp^a^, Cy^a^
Theta	*FcGSTT1*	FCD_00026472	6368	1311	437	49.02	9.07	Cp^a^, Cy^a^
EF1B *γ*	*FcEF1Bγ1*	FCD_00014039	3098	750	250	27.62	9.48	Cy^a^, Cp^b^

**Notes.**

cDNA (bp) complementary DNA CDS (bp) coding DNA Sequence MW Molecular Weight bp base pair aa amino acid kDa kilodalton Cy Cytoplasm Cp Chloroplast Mt Mitochondria Nu Nucleus

a Localization prediction by WoLF PSORT (https://www.genscript.com/wolf-psort.html).

b Localization prediction by BUSCA (http://busca.biocomp.unibo.it/).

### Chromosome localization and gene collinear relationship analysis

Fifty-three *FcGST* genes were unevenly dispersed on all 13 fig chromosomes (Fig. 2A). Fourteen members (26.42%) were densely mapped on chromosome 9. Chromosome 6 contained eight *FcGST* genes, followed by chromosome 10, 11, and 3 with six, five, and four members, respectively. Chromosomes 2, 5, and 8 had three genes each, while chromosome 7 and 13 had two genes each. Chromosomes 1, 4, and 12 contained only one *FcGST* gene each. We also found that members of the same subfamily were mostly grouped together. Thirty-nine GSTU genes were present in eight clusters, including three two-gene clusters, two three-gene clusters and one five-, eight- and 14-gene cluster. Four GSTF genes were located in a single one-gene cluster.

Gene family duplication investigation showed that the fig genome contained a total of 13 duplicated *FcGST* gene pairs (Fig. 2A). A maximum of five duplicated GST genes were located in chromosome 6, followed by three in chromosome 9 and chromosome 10, and one in chromosome 11. Out of the 13 gene pairs, 12 pairs were found as tandemly duplicated, and one pair appeared segmentally duplicated. Of the tandem duplications, ten pairs were in the tau subfamily and two pairs were in the phi subfamily, indicating that the tandem events contributed to these two subfamilies’ expansion. The average sequence similarity of 13 gene pairs was 66.27% (Fig. 2B), and gene pairs of the phi subfamily (FcGSTF3/4, and FcGSTF4/5) showed high protein sequence similarity, implying that the potential roles of these gene pairs might be similar.

**Figure 3:**
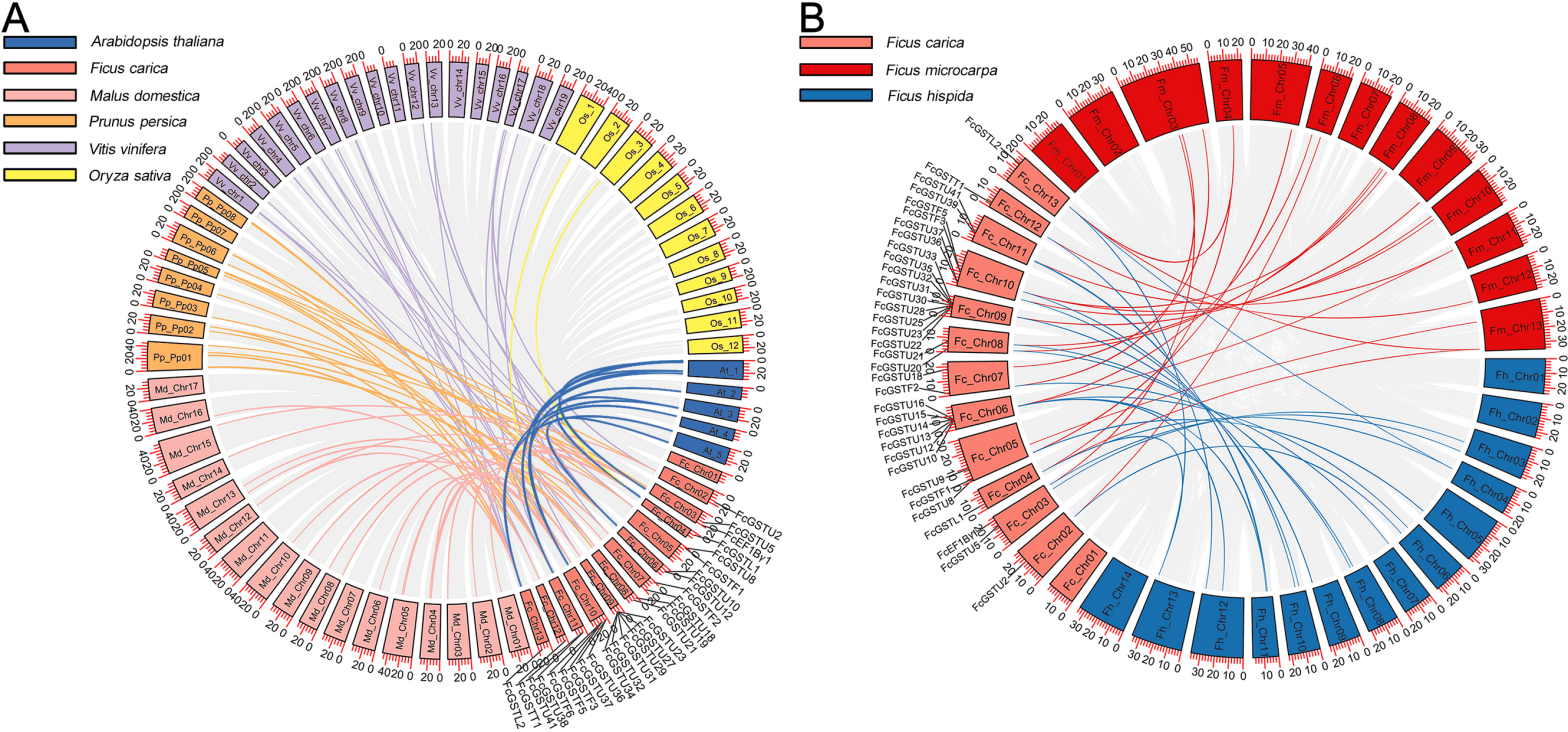
Collinear relationship of *GST* genes. (A) Syntenic analysis of *FcGST* genes with *Arabidopsis*, apple, peach, grape, and rice. Green, pink, orange, purple, and yellow curves represent homologous gene pairs between fig/*Arabidopsis*, fig/apple, fig/peach, fig/grape, and fig/rice, respectively. (B) Collinear relationship of *FcGST* genes in *Ficus* genus. Red, and blue curves represent homologous gene pairs between fig/*F. microcarpa*, and fig/*F. hispida*, respectively. Gray lines represent collinear gene pairs among fig with other specie genomes.

A synteny map was constructed to further estimate the homologous evolutionary relationship among GSTs of fig, the model species (*Arabidopsis* and rice), as well as three horticulture crops (apple, peach, and grape) (Fig. 3A). A total of 86 orthologous gene pairs were identified between fig and the five other examined species (Fig. 3A; Data S4). A maximum of 25 orthologous GST gene pairs were found between fig and peach, followed by fig and grape (24), and fig and apple (23). Twelve orthologous gene pairs were identified between fig and *Arabidopsis*, whereas only two orthologous gene pairs were found between fig and rice. We also investigated the collinear relationship of *GST* genes in the *Ficus* genus, including *F. carica*, *F. hispida*, and *F. microcarpa* (Fig. 3B). The collinearity analysis showed that 34 orthologous gene pairs existed between fig and *F. hispida*, and 33 orthologs between fig and *F. microcarpa* (Fig. 3B; Data S4).

Among these gene pairs, FcGSTL1 showed a syntenic relationship with those in the other seven species (Data S4). Eight FcEF1B*γ*1 and 13 FcGSTL2 collinear pairs were commonly identified between fig and all six dicot plants (Data S4). The results implied that these gene pairs may have formed before the ancestral divergence. However, a total of 13 FcGST genes were not identified by their orthologs between fig and the other seven species (Data S4), implying that these 13 members may be unique in fig evolution.

### Gene structure and conserved motif analysis of *FcGSTs*

**Figure 4:**
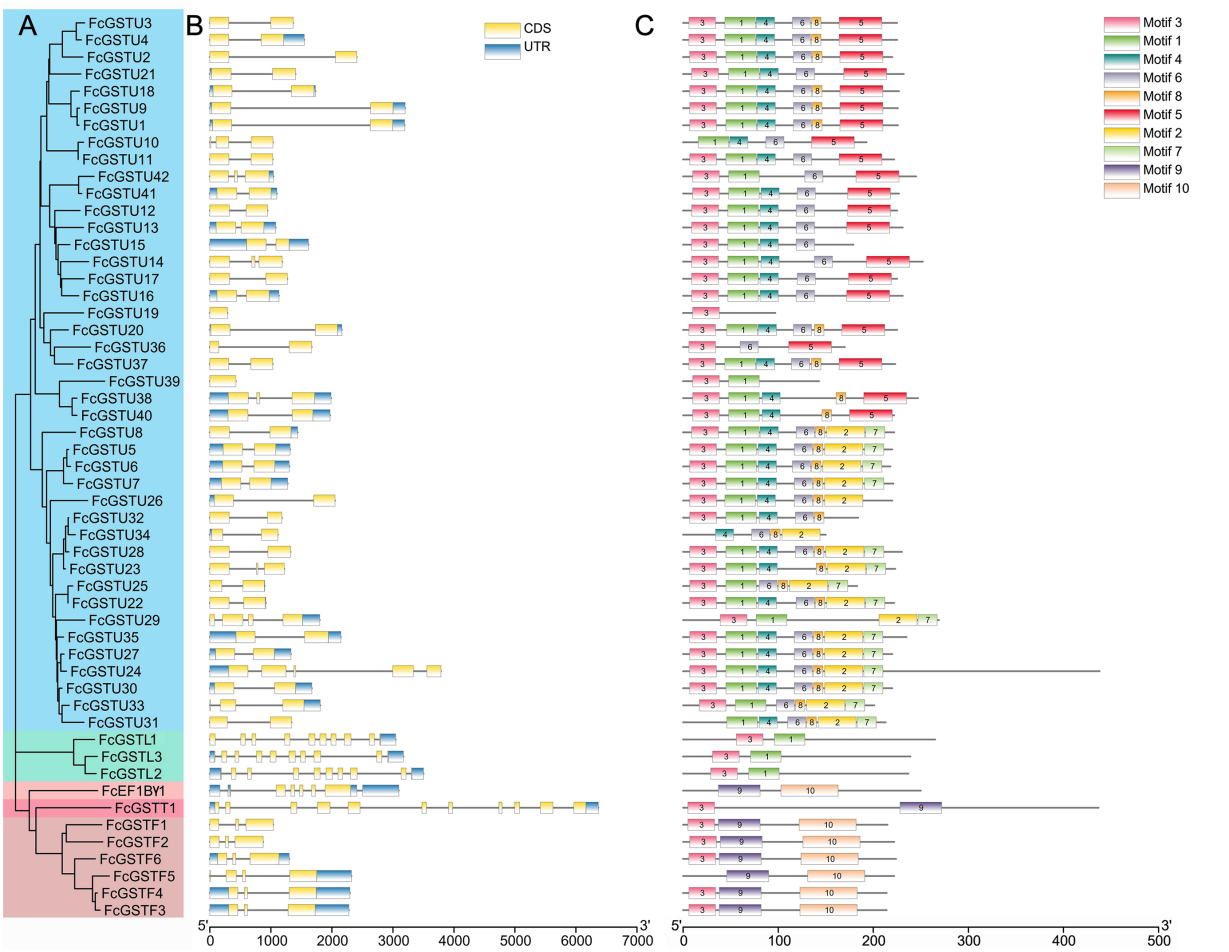
Phylogenetic relationships, gene structures, and conserved motifs of FcGSTs. (A) The phylogenetic tree of 53 FcGST family members. FcGSTs were divided into five subfamilies and marked with different colors. (B) Exon-intron organization of *FcGST* genes. Exons are shown as yellow rectangles; introns are shown as black lines, and the untranslated regions (UTR) are shown as blue rectangles. (C) Composition and distributions of conserved motifs in FcGST proteins. The conserved motifs are represented by different numbers and rectangle colors.

As depicted in Fig. 4B, *FcGST* genes contained one (*FcGSTU19* and *FcGSTU39*) to 11 (*FcGSTT1*) exons and showed similar exon-intron organization within the same subfamily, which was consistent with the phylogenetic analysis and data from other species (Figs. 4A and 4B; Data S5). A total of 207 (74.19%) *GST* genes of the tau family typically contained two exons in *Arabidopsis* (Sappl et al., 2004), rice (Jain, Ghanashyam & Bhattacharjee, 2010), apple (Jiang et al., 2019), *M. truncatula* (Hasan et al., 2021), tomato (Islam et al., 2017), and melon (Wang et al., 2020), and 32 of 42 (76.19%) *FcGSTUs* genes also contained two exons (Fig. 4B; Data S5). Sixty-three (86.30%) phi *GST* genes contained three exons, and the second exon was highly conserved in all seven plants, with a length of 48 nucleotides. There were 15 (68.18%) *GSTL* genes with 10 exons in *Arabidopsis*, apple, *M. truncatula*, tomato, melon, and fig. Among the exons of these *GSTL* genes, exons with lengths of 60, 86, 98, 102, 76, 107, and 80 were conserved and showed the same order. We identified several exons in the *FcEF1Bγ1* and *FcGSTT1* families that were conserved with other species, suggesting that these exons are crucial for typical domain composition.

To search the conserved amino acid motifs among FcGST proteins, the MEME online tool was utilized. Ten distinct motifs were identified in 53 FcGST proteins, ranging from 11 (motif 8) to 61 (motif 10) amino acids (Data S6). Except for FcGSTU10/34/31, FcEF1B*γ*1, and FcGSTF5, the remaining members all contained motif 3 (Fig. 4C). The rest of the motifs exhibited similar type and order within the same subfamily. For example, all six FcGSTF members contained motifs 3, 9, and 10, and motifs 1 and 3 were only present in FcGSTLs without the other motif. In the N-terminal of most of the FcGSTU members, the motif shared common conserved configuration. In the C-terminal, these genes could be separated into two groups according to motif 5, and motif 2 and 7, which was consistent with phylogenetic clade separation in the tau subfamily. We also observed that motifs 2, 4, 5, 6, 7, and 8 were specific to the tau subfamily.

### Protein structure prediction of GST families in fig

The secondary structure of 53 FcGST proteins were predicted using NPS@: SOPMA. The results showed that the presence of *α*-helix occupied a large position (51.36%), followed by random coils (29.86%), extended *β* strands (13.49%), and *β* turn (5.30%) (Data S7). We observed that the members within the same FcGST subfamily possessed similar protein sequences and showed similar secondary structure. Compared with phi family members, more *α*-helix (54.00%) and fewer random coils (28.13%) and *β* strands (12.92%) presented in the tau family composed the secondary structure with the exception of FcGSTU19/24/39 (Data S7). Members of lambda showed fewer *β* strands (10.45%) and *β* turns (3.78%) than FcGSTF proteins.

**Figure 5:**
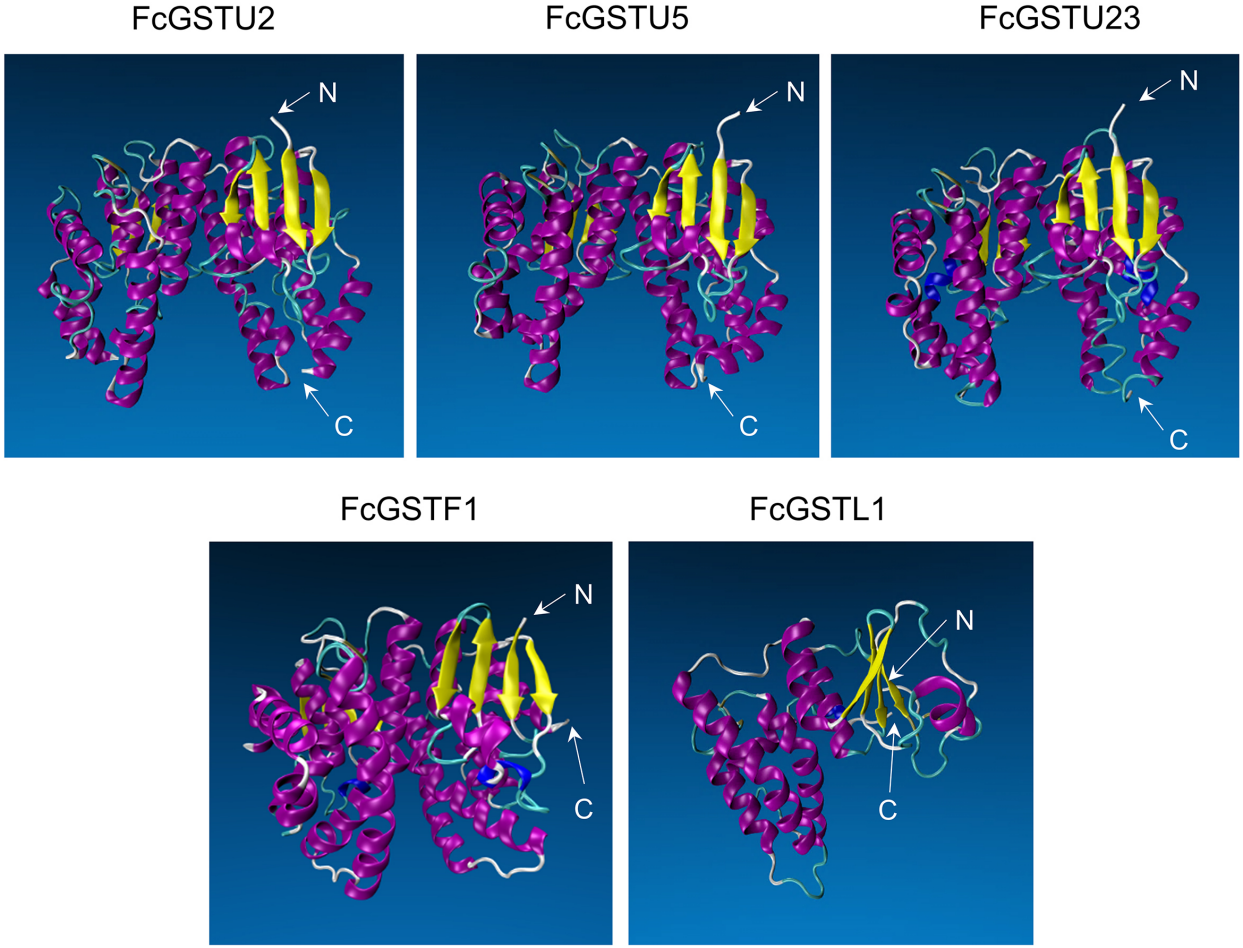
Tertiary structure models of five FcGST proteins. Tertiary structures of the five fig GST proteins using Swiss-model online software. Different colors indicate different types of structures. Purple, yellow, cyan, and white represent *α* helices, *β* sheets, turn, and random coil, respectively.

Tertiary structure models of five FcGST proteins were predicted by using Swiss-Model (Fig. 5). Each FcGST protein shared over 45% sequence identity with its template (Data S8). VERIFY3D showed that over 86% of the residues of these proteins had an average 3D-1D score ≥ 0.2 (Data S8). The ERRAT score showed an overall quality factor of over 92 (Data S8). Ramachandran plot results showed that the residues in the most favored regions of five proteins were more than 90% (Data S9), indicating that high quality models were built. Corresponding with secondary structure prediction, the *α*-helix was the major protein structure in 3-D models of all these five FcGSTs, followed by the random coil, *β* sheet, and turn (Fig. 5). The *α*-helix mainly presented in the C-terminal domain, which facilitated formation of substrate binding site, whereas the N-terminal domain composed of *α*-helix and *β* sheets (Fig. 5; Liu et al., 2013; Song et al., 2021a). The GST domains of three GSTU proteins shared the same conformation of the tertiary structure (Fig. 5).

### GO term, KEGG pathway enrichment, and PPI network analysis

**Figure 6:**
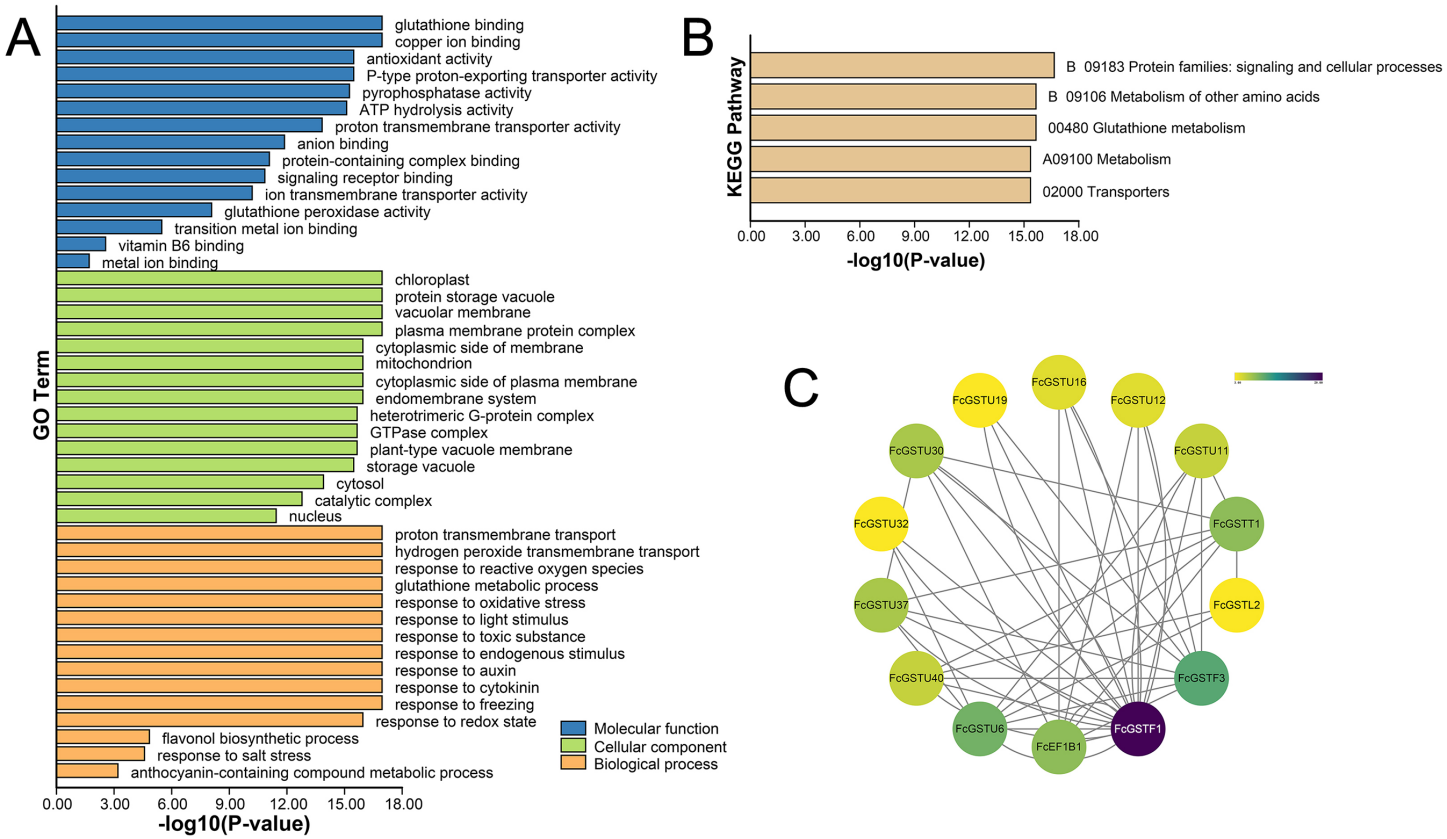
Functional annotation of FcGSTs. (A) GO function annotation of FcGST proteins. (B) KEGG enrichment of FcGST proteins. (C) Protein-protein interactions networks of FcGST members.

GO and KEGG annotations of FcGSTs were performed by BLASTP with Swiss-UniProt database and KofamKOALA web tool, respectively. GO enrichment analysis and KEGG enrichment analysis were conducted using TBtools (Chen et al., 2020). GO classification showed that FcGST proteins were mapped to one or more GO terms (Fig. 6A). *FcGSTs* were mainly enriched in terms of glutathione binding (94.34%), antioxidant activity (73.58%), anion binding (94.34%), and metal ion binding (69.81%) in the main GO category of “molecular function (MF)”. In “cellular component (CC)”, chloroplast (98.1%), vacuolar membrane (77.4%), mitochondrion (98.1%), endomembrane system (98.11%), cytosol (100%), and nucleus (100%) were largely enriched. Response to oxidative stress (100%) was the largest subcategory in “biological processes (BP)”. Glutathione metabolic process (98.11%), response to light stimulus (77.36%), toxic substance (98.11%), endogenous stimulus (96.23%), auxin (92.45%), cytokinin (64.15%), freezing (67.92%), and salt stress (33.96%) were also mainly enriched in BP. Moreover, we observed that processes related to flavonol (GO:0051555) and anthocyanin (GO:0046283) metabolism were enriched in BP. Additionally, the KEGG enrichment results showed that FcGST proteins mainly grouped into signaling and cellular processes, metabolism of other amino acids, glutathione metabolism, metabolism, and transporters, which was consistent with the KEGG enrichment result of CmGST in Hami melon (Fig. 6B; Song et al., 2021a). Protein-protein interactions (PPI) regulated approximately all cellular activities, including adjusting metabolic pathways in plants (Faraji, Rasouli & Kazemitabar, 2018). An interaction network among different members of FcGST proteins was constructed using the STRIING database. PPI network analysis showed that FcGSTF1 shared a high interaction degree with other FcGST members, which was associated with nine GSTU genes and FcEF1B*γ*1. FcGSTF3 showed links with eight GSTU genes and FcEF1B*γ*1 (Fig. 6C). PPI results indicated that FcGSTF1 and FcGSTF3 may implement key roles in the regulation mechanisms among FcGSTs in fig.

### *Cis*-acting elements in promoter regions of *FcGST* genes

**Figure 7:**
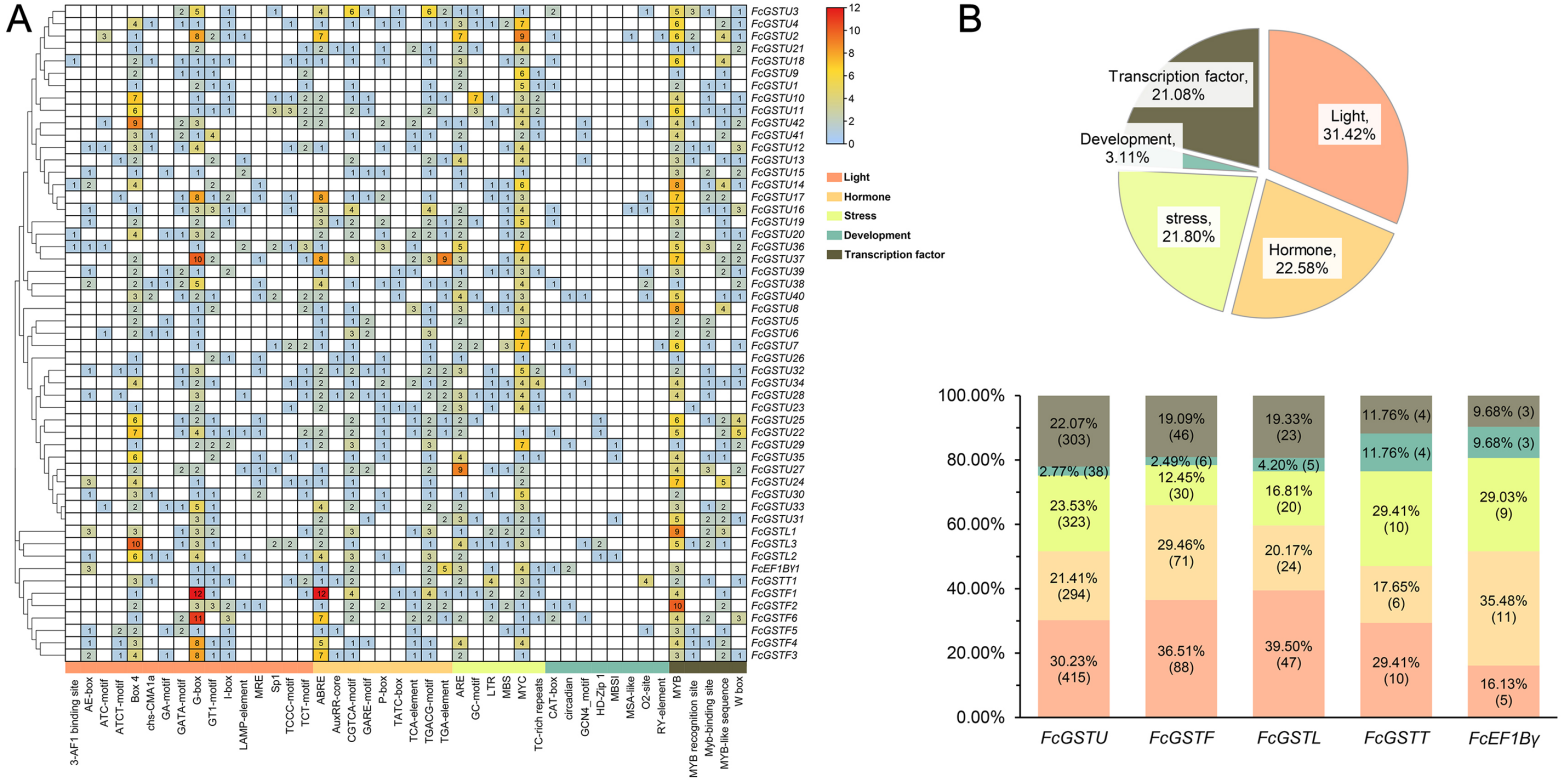
Analysis of the putative promoter of *FcGST* genes. *Cis*-acting elements of *FcGST* genes are classified into five groups: light, hormone, stress, development, and transcription factor. (A) Heatmap of *cis*-acting elements in the promoter of *FcGST* genes. The 2-kb upstream of the translation site was obtained for analysis, and the different colors and numbers represent the quantity of *cis*-acting elements in the promoter region of each *FcGST* gene. (B) Proportion of each group of *cis*-acting elements, and the proportion and number of different groups in the five subfamilies of *FcGSTs*.

To understand the transcriptional characteristics of *FcGST* genes, the putative *cis*-elements of 53 *FcGST* genes promoter regions located 2,000 bp from the upstream of the translation start site (ATG) were retrieved and scanned through the PlantCARE database. Forty-four kinds of *cis*-acting elements were predicted in the promoter region of 53 *FcGST* genes (Fig. 7). These elements were classified into five groups: light, hormone, stress, development, and transcription factor (Fig. 7A). Detailed information about the *cis*-acting elements is provided in Data S10. Approximately 31.42% elements responded to the light (Fig. 7B), and these elements were detected in the promoter region of all *FcGST* genes, ranging from four to 19 (Fig. 7A), suggesting that light stimulation is an important factor in the regulation of *FcGST* gene expression. The hormone responsive element was the second largest group in the subfamilies of tau (21.41%), phi (29.46%), and lambda (20.17%), but not the EF1B*γ* and theta subfamilies (Fig. 7B). Among these hormone-related elements, a total of 144 MeJA (methyl jasmonate) response elements (CGTCA-motif and TGACG-motif) were detected in 42 *FcGSTs*, followed by 122 abscisic acid response elements (ABRE) in 43 *FcGSTs*, 54 gibberellin response elements (GARE-motif, P-box, and TATC-box) in 36 *FcGSTs*, 48 auxin response elements (AuxRR-core and TGA-element) in 26 *FcGSTs*, and 28 salicylic acid responsive (TCA-element) elements in 20 *FcGSTs* (Fig. 7A). The upstream region of all 53 *FcGSTs* possessed stress-related elements, including anaerobic induction (ARE, and GC-motif), low-temperature response elements (LTR), drought-response elements (MBS, and MYC), and defense and stress (TC-rich repeats) (Fig. 7A). By contrast, only 3.11% *cis*-acting elements related to plant development were identified, including CAT-box (15), circadian (8), GCN4_motif (7), HD-Zip 1 (5), MBSI (4), MSA-like (2), O2-site (13), and RY-element (2) (Figs. 7A and 7B). Additionally, several transcription factor- binding elements were also detected in *FcGST* promoters, such as MYB, Myb-binding site, MYB-like sequence, MYB recognition site, and W box (WRKY-binding motif), which may be involved in the direct regulation of these *FcGST* transcriptions (Fig. 7A).

### Expression pattern of *FcGSTs* during fig fruit ripening

**Figure 8:**
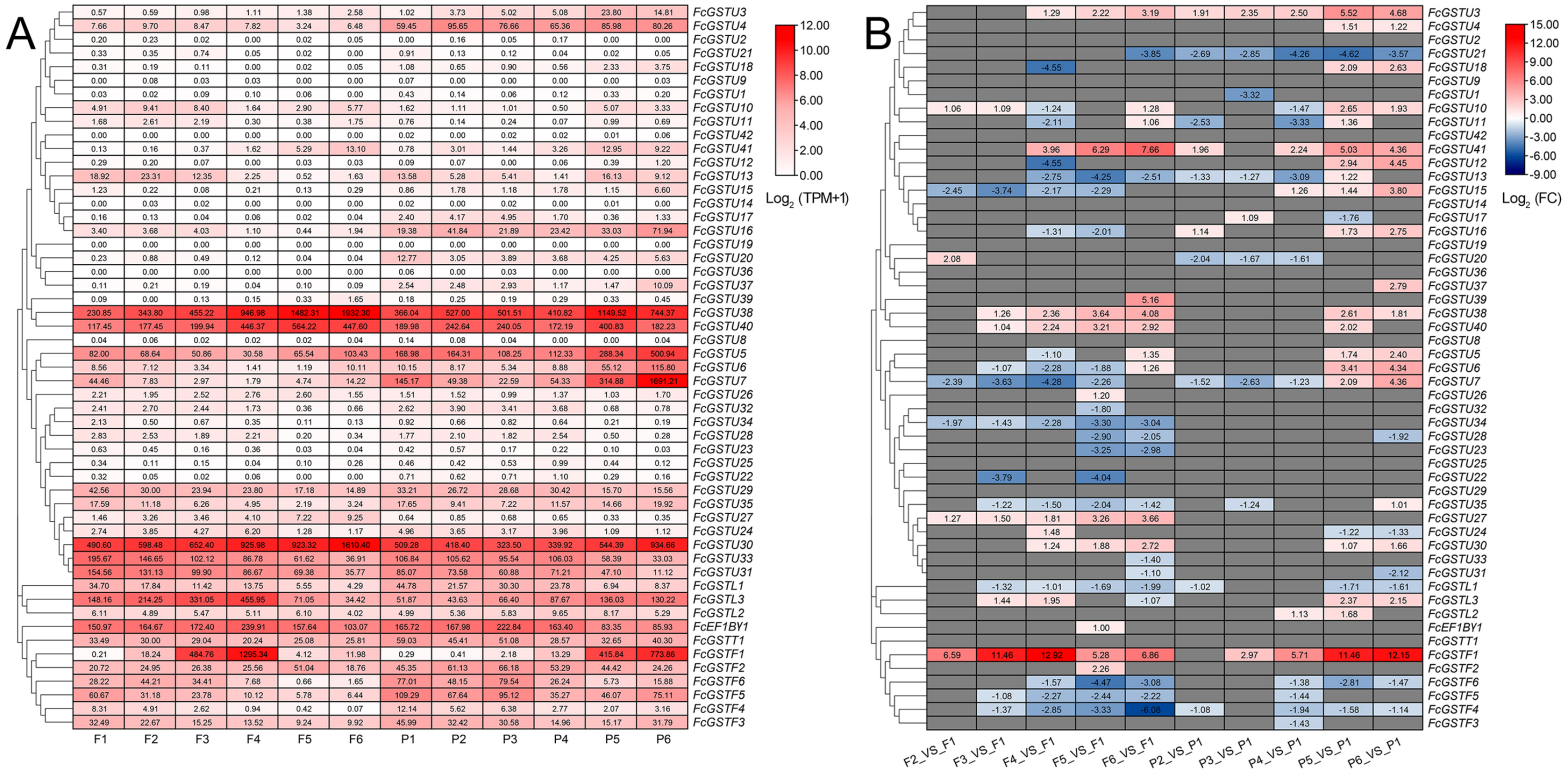
Heatmap of expression profiles for *FcGST* genes in the female flower and peel of fig fruit. (A) Transcript abundance of *FcGST* genes in six stages of female flower tissue and peel. Expression levels are plotted in different shades of red based on Log _2_-transformed (TPM+1). The number in each rectangle represents the TPM value calculated by StringTie quantify. (B) Relative expression levels of *FcGST* genes during fig fruit ripening. The relative expression levels of each *FcGST* gene are expressed by different shades of red (up-regulation) or blue (down-regulation) and the log _2_FC value. The genes with no change are indicated with grey. F1-F6 and P1-P6 indicate the six development stages of fig female flower tissue and peel, respectively.

To comprehensively illustrate *FcGST* gene expression characteristics, we investigated these gene expression pattern on three published RNA-seq data, including six stages of female flower and fruit peel during ‘Zibao’ fig fruit development (Fig. 8; Zhai et al., 2021), the fruit peel of ’Green Peel’ and ’Purple Peel’ at young and mature stages (Data S11A; Wang et al., 2017), and female flowers after ethephon application at two, four, and six days (Data S11B; Cui et al., 2020).

During fig fruit development, most *FcGSTU* genes showed slight or undetectable expression (Fig. 8, Data S11A), even under ethephon treatment (Data S11B). However, *FcGSTU30/38/40* expressed highly in both female flower and fruit peel, and the expression abundance significantly increased in fruit maturation (Fig. 8, Data S11B). However, the difference was not observed in mature fruit peel between ‘Green Peel’ and ‘Purple-Peel’ (Data S11A). *FcGSTU5/6/7* showed similar expression trends in fruit peel, which slightly declined to the bottom at P4 but rapidly increased in the mature stage (P5 and P6), and was especially highly expressed in the mature fruit peel of ‘Purple-Peel’ (Fig. 8, Data S11A). The specific expression changes of *FcGSTF1* were noticeable. *FcGSTF1* was significantly up-regulated until F4 and then suddenly decreased in F5 and F6, whereas its expression was continually upregulated in fruit peel (Fig. 8). Compared with ’Green Peel’, *FcGSTF1* exhibited high expression abundance in the mature fruit peel of purple peel (Data S11A), which completely matched with the fruit peel phenotype (Wang et al., 2017). Additionally, we observed that several genes also increased in response to ethylene induction, such as *FcGSTU5/6/7/38/40* after two d of ethephon treatment, and *FcGSTU30* and *FcGSTF1* at six d. Previous study results showed that ethephon did not accelerate anthocyanin accumulation but instead inhibited pigmentation in fig flower (Cui et al., 2020), which was highly consistent with the expression changes of anthocyanin biosynthetic genes (*F3H*, *DFR*, and *LDOX*) (Cui et al., 2020), *FcGSTF1*, and *FcGSTU5/6/7* (Data S11B). These results showed that *FcGSTF1* and *FcGSTU5/6/7* expression was related to the development of anthocyanins, and we conjecture that these genes may play additional roles in the coloration of fig fruit.

### Anthocyanin accumulation-related *GSTs* gene features and the expression assay in fig fruit peel

**Figure 9:**
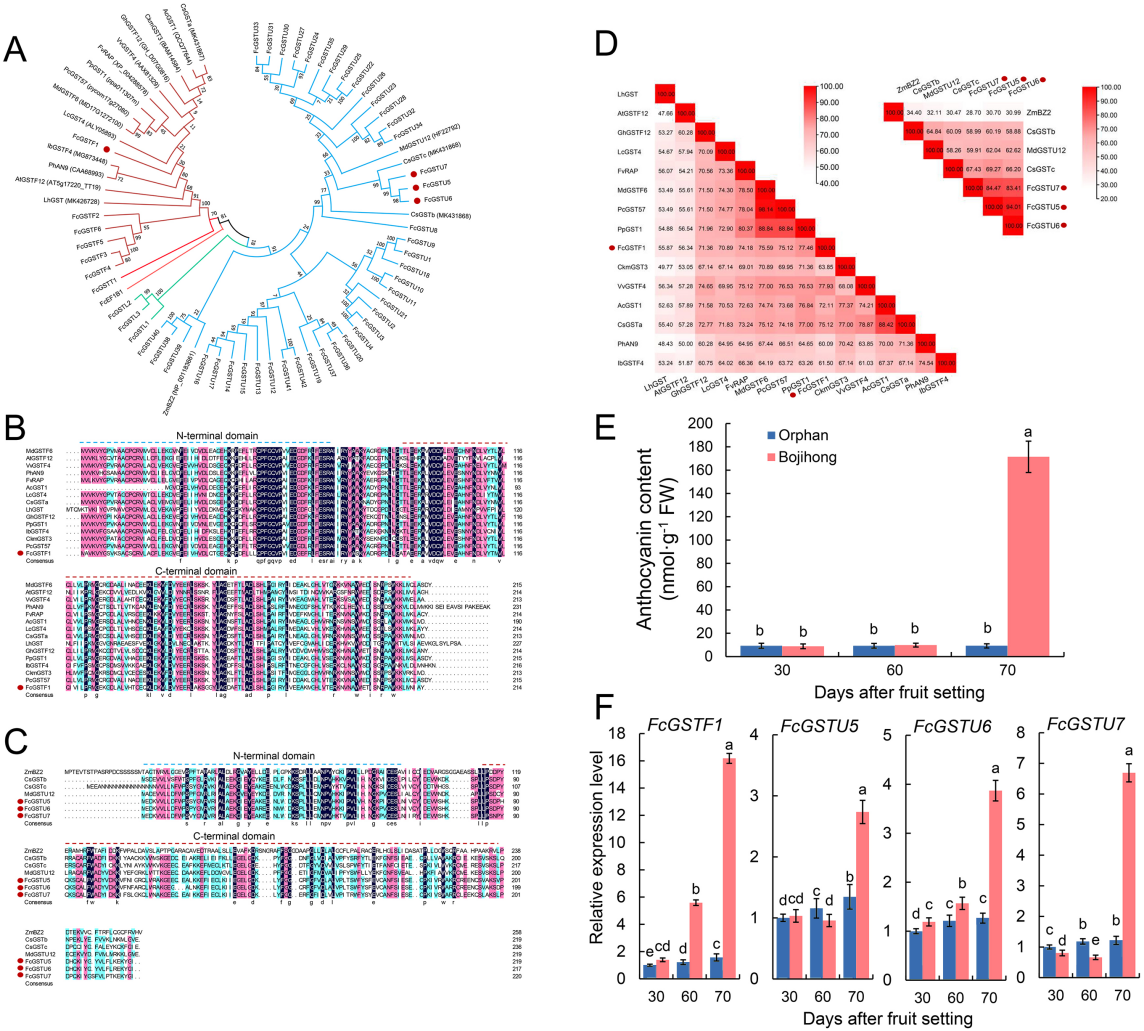
Function characterization of *FcGST* genes related to anthocyanin accumulation in fig fruit peel. (A) Phylogenetic analysis of the function of FcGSTs and several GST proteins in anthocyanin transport. (B) Protein sequence alignment of FcGSTF1 and other anthocyanin transport-related GSTs belonging to the phi subfamily. (C) Protein sequence alignment of FcGSTU5/6/7 and other anthocyanin transport-related GSTs belonging to tau subfamily. The dotted blue and red lines indicate the N- and C-terminal domain, respectively. (D) Identity/similarity analysis of amino acid sequences of FcGSTF1 with other anthocyanin transport-related GSTFs, and FcGSTU5/6/7 with ZmBZ2, CsGSTb, MdGSTU12, and CsGSTc. The different shades of red represent these sequences’ similarity degree. (E) The anthocyanin content in the fruit peel of ’Orphan’ and ‘Bojihong’ 30, 60, and 70 days after fruit setting. (F) Expression profile of *FcGSTF1* and *FcGSTU5/6/7* were detected in the fruit peel of ‘Orphan’ and ‘Bojihong’ 30, 60, and 70 days after fruit setting. Gene relative expression was normalized to the expression level of ‘Orphan’ fruit peel at 30 days after fruit setting. Different lowercase letters indicate significant difference (*p* < 0.05).

Furthermore, we investigated the gene features and expression patterns in the fruit peel of two common fig cultivars during the fruit ripening. Multiple sequence alignment and phylogenetic analyses with other reported anthocyanin-related GST genes were performed. The results showed that FcGSTF1, CsGSTa, AcGST1, CkmGST3, GhGSTF12, VvGSTF4, FvRAP, PpGST1, PcGST57, MdGSTF6, LcGST4, IbGSTF4, PhAN9, AtGSTF12, and LhGST were clustered together (Fig. 9A). FcGSTU5/6/7 were found to be closely associated with anthocyanin-related tau family members, such as MdGSTU12, CsGSTc, and CsGSTb (Fig. 9A). Sequence alignment revealed that FcGSTF1 shared the same conserved domain and showed a high sequence similarity (69.10% on average) with anthocyanin-related GSTF genes (Figs. 9B and 9D). FcGSTU5/6/7 sequences shared similar protein sequences and 61.52%, 67.63%, and 59.35% similarity with MdGSTU12, CsGSTc, and CsGSTb, respectively (Figs. 9C and 9D). Furthermore, we investigated the anthocyanin content alteration and gene expression levels of *FcGSTF1* and *FcGSTU5/6/7* in two common fig cultivars with different fruit peel colors (Figs. 9E and 9F). Compared with the green-yellow fig fruit peel of ‘Orphan’, *FcGSTF1* and *FcGSTU5/6/7* were significantly induced in the red-purple fruit peel of ‘Bojihong’, which may lead to rapid anthocyanin accumulation at 70 d after fruit setting (Figs. 9E and 9F). Among these genes, the expression of *FcGSTF1* was significantly higher than *FcGSTU5/6/7*, even at the fruit color turning stage (60 days after fruit setting) (Fig. 9F).

## Discussion

### *FcGSTF1* and *FcGSTU5/6/7* are associated with anthocyanin accumulation in fig peel

Anthocyanin is not only an important and beneficial pigments, it is also a key appearance trait of fruit coloration. Recently, several studies have characterized the molecular function of a series of anthocyanin biosynthetic genes during fig fruit coloration, including *FcPAL*, *FcC4H*, *Fc4CL*, *FcCHS1*, *FcCHS2*, *FcCHI*, *FcF3H*, *FcF3*′*H*, *FcDFR*, *FcANS*, and *FcUFGT* (Wang et al., 2017; Lama et al., 2020; Li, An & Wang, 2020). However, the function of *FcGST* is still unknown in fig, while its homologous genes have been verified as the anthocyanin transporter in various plant species (Kitamura, Shikazono & Tanaka, 2003; Sun, Li & Huang, 2012; Luo et al., 2018; Jiang et al., 2019). In our study, GO analysis showed that several *FcGST* family genes were enriched in the flavonol biosynthetic process (GO:0051555) and anthocyanin-containing compound metabolic process (GO:0046283) (Fig. 6A). During fig fruit development, anthocyanin accumulation initiation was earlier in female flower tissues than in fruit peel (Wang et al., 2021; Cui et al., 2020). The particular expression pattern of *FcGSTF1* was highly matched with the obvious space–time differences of the coloration process between female flower and fruit peel (Fig. 8; Data S11B). Additionally, FcGSTF1 showed high sequence similarity with the GSTs’ known function in anthocyanin transport (Figs. 9B and 9D) (Kitamura, Shikazono & Tanaka, 2003; Luo et al., 2018; Jiang et al., 2019). We also found that *FcGSTU5/6/7* were significantly induced in the mature stages (stage 5 and 6) of ‘Zibao’ fruit peel (Fig. 8; Data S11A), and showed closer relationships as well as similar protein sequences with anthocyanin-related tau family members (Figs. 9A, 9C and 9D) (Liu et al., 2019a; Liu et al., 2019b; Zhao et al., 2021). Moreover, high consistency between anthocyanin accumulation trends and expression level changes of *FcGSTF1* and *FcGSTU5/6/7* were also detected (Fig. 9). The results reveal that these genes may play important roles in fig fruit peel coloration, although the specific function of these genes in anthocyanin transport requires additional research.

In high plants, MYB is the crucial transcriptional factor (TF) of secondary metabolism, and is not only associated with expression level regulation of anthocyanin biosynthesis structural genes, but also the regulatory function in anthocyanin transport stage by binding to *GST* promoter (Hu et al., 2016). In *Arabidopsis*, overexpression of *PAP1* (a R2R3 MYB transcription factor) induced *TT19* up-regulation which contributed to anthocyanin accumulation (Wangwattana et al., 2008). Moreover, studies showed that MYB activates *GST* gene expression by binding to MYB binding sites (MBSs) in several horticultural plants, such as *LcMYB1* in litchi (Hu et al., 2016), *MdMYB1* in apple (Jiang et al., 2019), *AcMYBF110* in kiwifruit (Liu et al., 2019b), *CsMYB75* in purple tea (Wei et al., 2019a), *PpMYB10.1* in peach (Zhao et al., 2020), *LhMYB12-lat* in lilies (Cao et al., 2021), and *ScMYB3* in cineraria (Cui et al., 2021). Likewise, we also identified a total of 20 MBSs in *FcGSTF1* and *FcGSTU5/6/7* (Fig. 7; Data S10). Among these MBSs, several have been characterized as the MYB TF binding site in studies. In peach, trans-activation activity of *PpMYB10.1* was reduced by 72% when MBS1 (CAACCA) was mutated (Zhao et al., 2020). In apple, *MdMYB1* could directly bind to the *MdGSTF6* promoter with the MBS (CAACTG) (Jiang et al., 2019). Recently, *FcMYB114*, *FcMYB21*, and *FcMYB123* were found to play positive roles in fig fruit anthocyanin biosynthesis (Jiang et al., 2019; Li, An & Wang, 2020). Thus, we conjecture that MYB, including these MYB TFs, may make a major contribution to *FcGST* expression activity during fig fruit coloration. Interestingly, the first fig bHLH gene involved in fruit color development, *FcbHLH42*, showed highly similar expression changes with *FcGSTF1* in the six female flower tissue and fruit peel stages (Song et al., 2021b). The study confirmed that FcbHLH42 interacted with strawberry FaMYB10 to promote anthocyanin accumulation in transgenic tobacco (Song et al., 2021b). We hypothesize that FcbHLH42 may directly interact with FcMYB TFs in the regulation and expression of *FcGSTF1*. Future studies should illustrate the complex transcriptional regulation mechanism of *FcGSTF1* and *FcGSTU5/6/7-* mediated fig fruit peel coloration. Our results may give the insight into the GST gene family in fig and assist in understanding the roles of several key *FcGST* genes.

### Characteristics of *FcGST* gene family members in fig genome

GST is a large, ubiquitous, and ancient protein superfamily, that has been identified in various plant species (Sappl et al., 2004; Jain, Ghanashyam & Bhattacharjee, 2010; Dong et al., 2016). In this study, we identified a total of 53 *FcGST* gene members in fig, and classified them into five distinct subfamilies (Table 1). Among the different subfamilies, tau was the largest, followed by phi (Fig. 1), which supported their dominant distribution in other species, such as *Arabidopsis* (Sappl et al., 2004), rice (Jain, Ghanashyam & Bhattacharjee, 2010), and apple (Jiang et al., 2019). GST proteins are localized in various subcellular compartments, and they mainly show cytosolic localization (Raza, 2011). Likewise, most FcGST members were also predicted to profusely present in cytoplasm (Table 1). In plants, most cytosolic GSTs typically function as dimers with molecular weights ranging from 23 to 29 kDa (Frova, 2006), which is similar to GrGSTs (26.48 kDa) in *G. raimondii*, GaGSTs (26.64 kDa) in *G.arboreum* (Dong et al., 2016), CmGST (23.51 kDa) in Hami melon (Song et al., 2021a), and FcGSTs (26.25 kDa) in fig (Table 1).

Tandem, segmental, and whole-genome duplication are common genetic events that are responsible for gene family expansion and diversification in plants (Flagel & Wendel, 2009). Previous studies suggested that segmental gene duplication mainly occurred for the amplification of tau and phi subfamily *GST* genes in pear (Wang et al., 2018), tomato (Islam et al., 2017), pepper (Islam et al., 2019), chickpea (Ghangal et al., 2020), and *M. truncatula* (Hasan et al., 2021). However, 20 of the 48 *FcGSTU* and *FcGSTF* genes had tandem duplicate events (Fig. 2), similar to the ratio reported for Hami melon (14 out of 29) (Song et al., 2021a) and *A. thaliana* (22 out of 41) (Chi et al., 2011) GSTU and GSTF subfamilies. It appears that tandem repeat events mainly contributed towards the evolution of tau and phi subfamily *GST* genes in fig.

The conserved exon-intron organization could reveal information about the evolutionary relationship (Sánchez et al., 2003). In the same subfamily, a specific exon-intron arrangement pattern and several length-conserved exons were identified in fig and other plant species (Sappl et al., 2004; Jain, Ghanashyam & Bhattacharjee, 2010; Islam et al., 2017; Jiang et al., 2019; Wang et al., 2020; Hasan et al., 2021) (Fig. 4B; Data S5). Most FcGST members also shared a similar motif organization in the same subfamily (Fig. 4C). These results reveal that FcGST family members are evolutionarily conserved across distinct phylogenetic groups. Moreover, the members of eight different GST subfamilies showed high similarities between fig, *Arabidopsis*, and rice (Fig. 1), indicating the ancient evolution of these subfamilies before the split of monocots and dicots (Islam et al., 2017; Islam et al., 2019; Wang et al., 2020).

### *FcGSTs* may play pleiotropic roles in fig

GST family genes show versatile functions in diverse plants (Vaish et al., 2020). GO, KEGG enrichment, and *cis*-acting element analyses reflected their multipurpose roles in fig (Figs. 6 and 7). A considerable amount of information has been gathered on various abiotic stress management roles of GST family genes in plants (Vaish et al., 2020). In general, the complex stress regulation roles of the GST gene family are highly correlated with reactive oxygen species (ROS) scavenging (Estévez & Hernández, 2020). Here, the molecular function of glutathione peroxidase activity (GO:0004602), biological process of ROS stress response (GO:0006979, GO:0000302, GO:0051775), toxic substance (GO:0009636), freezing (GO:0050826) and salt (GO:0009651), as well as the glutathione metabolism pathway (ko00480), were enriched (Figs. 6A and 6B). Furthermore, we also found that at least one stress responsive element was presented in the *FcGST* promoter region which might be associated with anaerobic stress, drought, and cold (Fig. 7). Therefore, further studies are needed to verify the positive roles of *FcGST* genes in the improvement of abiotic stress tolerance in fig.

GSTs are known to respond to multiple phytohormones, including auxin, cytokinin, MeJA, ABA, salicylic acid, ethylene, and other hormones (Hasanuzzaman et al., 2017). GO and promoter prediction analysis suggest that *FcGSTs* may function in various hormone-regulated fig tree growth and development (Figs. 6A and 7).

In plants, tau subfamily members might be involved in sugar signaling. Five tau genes in *Arabidopsis* and 26 tau GST members in sorghum were up-regulated under sucrose treatment (Chi et al., 2011). In pear, the expression of *PpGST2* in the tau subfamily was significantly increased by glucose during fruit development (Shi et al., 2014). In this study, the expression of *FcGSTU30/38/40* was developmentally upregulated in fig fruit flower and peel (Fig. 8; Data S11A). Moreover, the high consistency between the changes of *FcGSTU30* expression abundance and the cumulative increase of the soluble sugar content in fig flower after ethephon treatment were observed (Data S11B; Cui et al., 2020). These data revealed that *FcGSTU 30/38/40* may play a role in the sugar signaling pathway and ethylene-induced ripening in fig fruit.

## Conclusion

In this study, a total of 53 *GST* genes identified in the fig genome database were divided into five distinct subfamilies. The members of FcGST in the same phylogenetic subfamily showed evolutionary conservation with other plant species in the GST family. The *FcGST* family genes may play multiple functions in fig tree growth and development. Furthermore, our results suggest that *FcGSTF1* and *FcGSTU5/6/7* might play roles in regulating fruit peel coloration, but the specific molecular function and regulation mechanism still need to be verified by further study.

##  Supplemental Information

10.7717/peerj.14406/supp-1 Data S1 Protein sequences of 53 AcGSTs, 77 OsGSTs, and 53 FcGSTs

10.7717/peerj.14406/supp-2 Data S2 Primers used in this study.

10.7717/peerj.14406/supp-3 Data S3 Comparison of GST gene numbers among different species

10.7717/peerj.14406/supp-4 Data S4 Collinear relationship between fig and seven other species

10.7717/peerj.14406/supp-5 Data S5 Gene structure detail of fig and six other species

10.7717/peerj.14406/supp-6 Data S6 Putative motifs predicted in fig GSTs.

10.7717/peerj.14406/supp-7 Data S7 Secondary structural statistics of the 53 FcGST proteins

10.7717/peerj.14406/supp-8 Data S8 Validation of predicted 3D models.

10.7717/peerj.14406/supp-9 Data S9 Ramachandran plot analyses of three-dimensional model of five FcGST proteins

10.7717/peerj.14406/supp-10 Data S10 Detailed information of *cis.*-acting elements in *FcGST* promoter regions

10.7717/peerj.14406/supp-11 Data S11 The expression of *FcGST* in green peel and purple peel (A), and in female flower after 2, 4, and 6 d of ethephon treatment(B)

10.7717/peerj.14406/supp-12 Data S12 Raw data of anthocyanin content in the fruit peel of ‘Orphan’ and ‘Bojihong’ 30, 60, and 70 d after fruit setting

10.7717/peerj.14406/supp-13 Data S13 Raw data of qRT-PCR of *FcGSTF1/GSTU5/6/7.* in the fruit peel of ‘Orphan’ and ‘Bojihong’ 30, 60, and 70 d after fruit setting

## Additional Information and Declarations

### Data Availability

The following information was supplied regarding data availability: The raw data are available in the Supplemental Files.
