# Supplementary figures and images for: Genome-wide in silico identification of glutathione S-transferase (GST) gene family members in fig (Ficus carica L.) and expression characteristics during fruit color development
Source: PeerJ. 2023 Jan 25;11:e14406. doi: 10.7717/peerj.14406 (PMC9884035; doi:10.7717/peerj.14406)

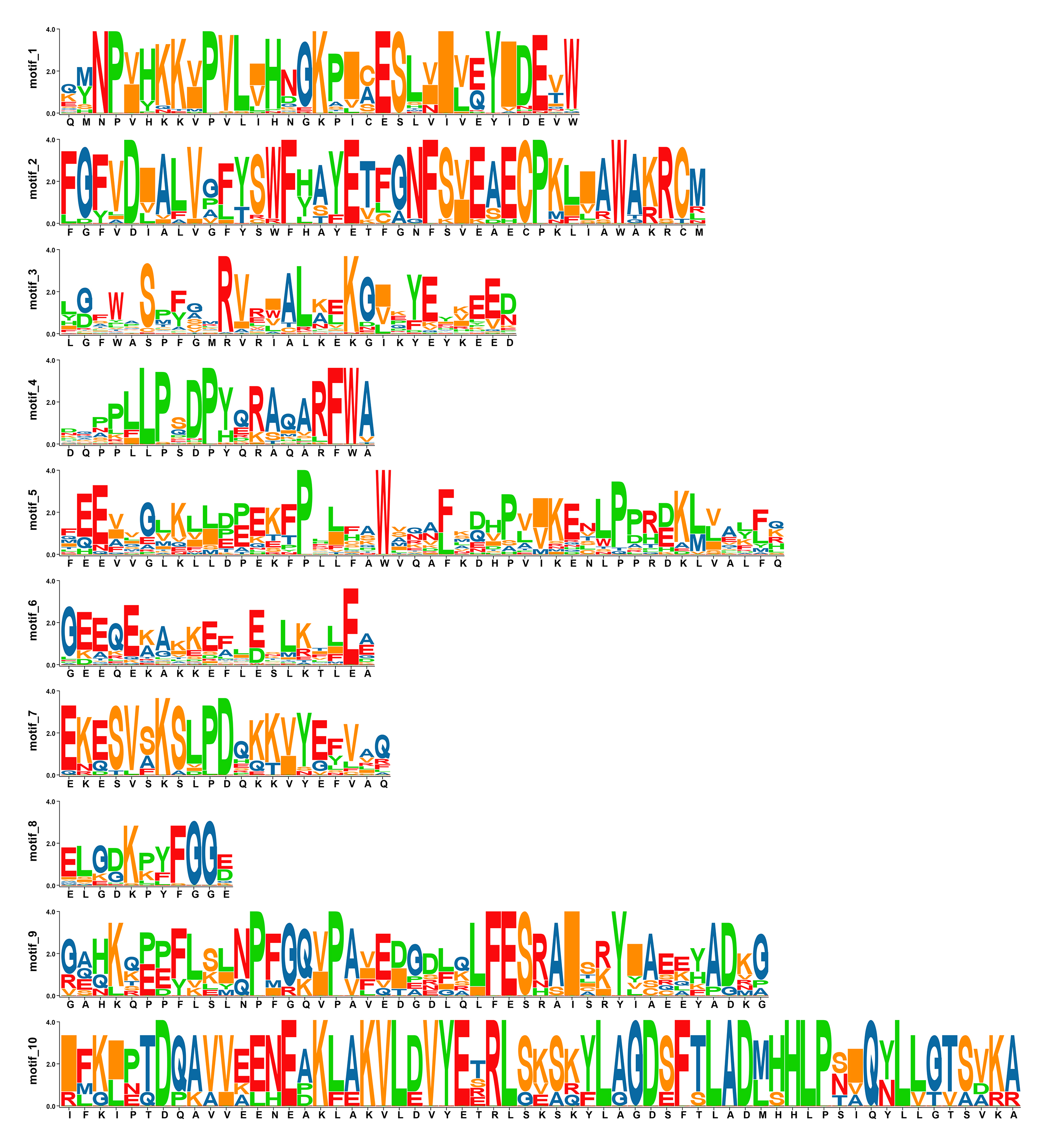

Supplement: Data S6 [file peerj-11-14406-s006.jpg]

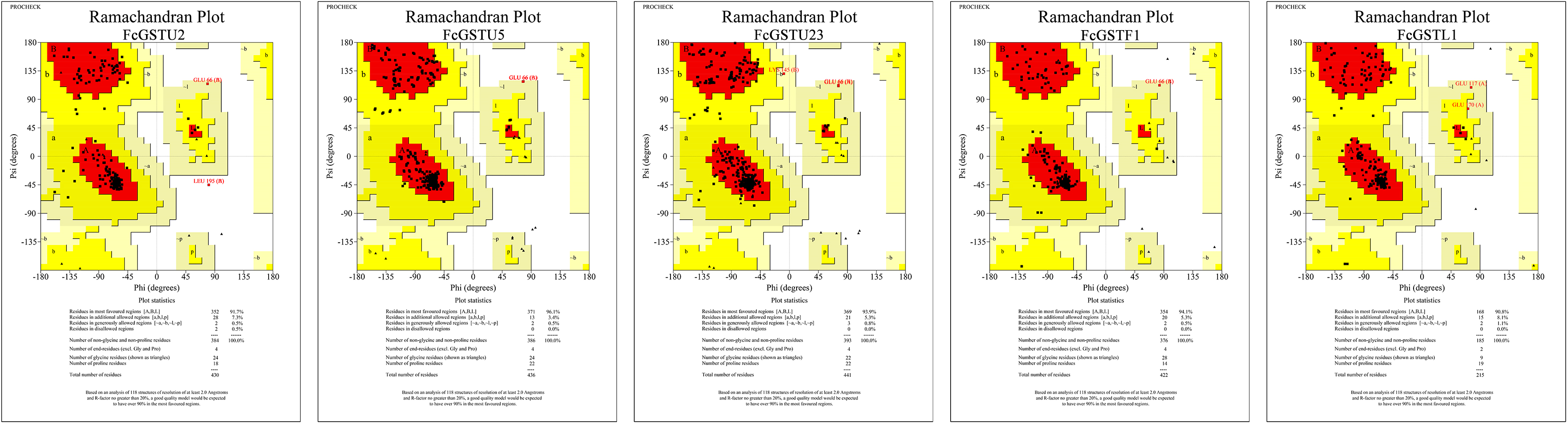

Supplement: Data S9 [file peerj-11-14406-s009.jpg]

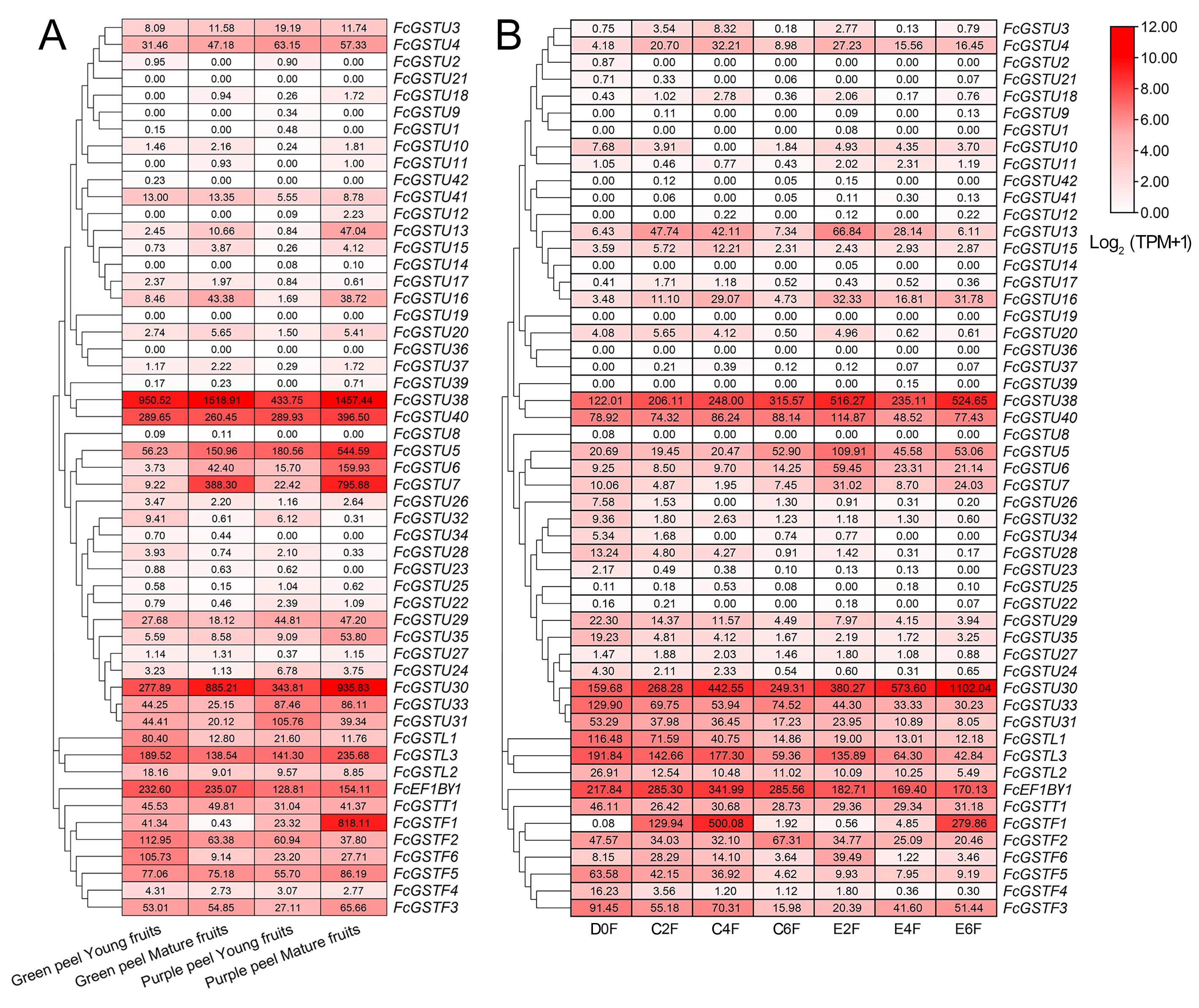

Supplement: Data S11 [file peerj-11-14406-s011.jpg]
